# Genomic landscape of lung cancer in the young

**DOI:** 10.3389/fonc.2022.910117

**Published:** 2022-09-29

**Authors:** Rossana Ruiz, Marco Galvez-Nino, Katia Roque, Jaime Montes, Maria Nuñez, Luis Raez, Sergio Sánchez-Gambetta, Sandra Jaúregui, Sandra Viale, Edward S. Smith, Joseph A. Pinto, Luis Mas

**Affiliations:** ^1^ Departamento de Oncología Médica, Instituto Nacional de Enfermedades Neoplásicas, Lima, Peru; ^2^ Departamento de Patología, Instituto Nacional de Enfermedades Neoplásicas, Lima, Peru; ^3^ Memorial Cancer Institute/ Memorial Health Care System, Florida International University (FIU), Miami, FL, United States; ^4^ Roche Farma, Lima, Peru; ^5^ Centro de Investigación Básica y Traslacional, Auna Ideas, Lima, Peru

**Keywords:** lung cancer, non-small cell lung cancer, genomic profiling, genomic alterations, tumor mutational burden

## Abstract

**Background:**

Lung cancer in the young is a rare entity of great interest due to the high frequency of targetable mutations. In this study, we explored the genomic landscape of non-small cell lung cancer (NSCLC) in young patients and compared it with genetic alterations in older patients.

**Methods:**

Comparative study of the genomic profile of NSCLC young (≤40 years old) vs older patients (>40 years old) from Instituto Nacional de Enfermedades Neoplásicas (INEN) in Lima, Peru. Archival paraffin-embedded tumor samples were profiled with FoundationOne CDx assay to identify short variants alterations (insertions and deletions), copy number variations (CNV), tumor mutational burden and microsatellite instability in 324 driver genes and rearrangements in 28 commonly rearranged genes. A targetable alteration was defined as any alteration in a driver oncogene for which an FDA approved therapy existed at the time of study enrollment.

**Results:**

Overall, 62 tumors were profiled, 32 from young and 30 from older patients. All clinicopathological features (smoking status, clinical stage, and histology) were similar between groups, except for gender (65.6% of females in the younger group vs 40% in the older group, P=0.043). At least one actionable mutation was present in 84.4% and 83.3% in younger and older patients, respectively. Alteration rates in the main genes were: BRAF, 3.1%(n=1) vs 0%; EGFR, 46.9% (n=15) vs 43.3% (n=13); ERBB2, 12.5% (n=4) vs 16.7% (n=5); KRAS, 15.6% (n=5) vs 16.7% (n=5); ALK, 6.3% (n=2) vs 3.3% (n=1); RET, 0.0% vs 3.3% (n=1); ROS1, 3.1% (n=1) vs 3.3% (n=1); NTRK1, 0.0% vs 3.3% (n=1) and MET, 3.1% (n=1) vs 13.3% (n=4). Mean TMB was 4.04 Mut/Mb (SD ± 3.98) for young vs 8.06 Mut/Mb (SD ± 9.84) for older patients (P=0.016). There were not significant differences in CNV, frequency of gene rearrangements, or microsatellites instability.

**Conclusion:**

NSCLC in the young in our cohort was characterized by a high frequency of actionable genetic aberrations and a low TMB, which was also true for our older patients. The enrichment of actionable mutations in young patients described in other reports might be attributed to differences in the etiology and clinicopathological characteristics between younger and older patients and therefore not be applicable to all populations.

## Highlights

- Clinicopathological characteristic between younger and older patients were similar, importantly most of them were never smokers.
- NSCLC in the young in our cohort was characterized by a high frequency of actionable genetic aberrations and a low TMB.
- No differences were found in the rate or distribution of actionable mutation between younger and older patients (84.4% vs 83.3%, respectively).
- The enrichment of actionable mutations in young patients described in other reports might be attributed to differences in the etiology and clinicopathological characteristics between younger and older patients and therefore not be applicable to all populations.

## Introduction

Lung cancer in young people is considered a unique entity due its clinicopathological features, oncogenesis, and prognosis (1). These characteristics could be related to a different exposition to environmental risk factors such as tobacco, occupational carcinogens, or air pollution, as well as the presence of age-related genetic alterations (2, 3).

Young patients with lung cancer represent 2-3% of lung cases worldwide, however, this may vary depending on the age threshold used and the country reported. We previously reported that lung cancer in patients younger than 40 years is more frequently diagnosed at advanced stages in never-smoker women (4).

Large genomic projects, such as The Cancer Genome Atlas (TCGA), identified an array of actionable mutations, which opened new therapeutic approaches for NSCLC and changed the prognosis of this disease. Geographical and age-related variations have been reported for these gene mutations. Likewise, it has been reported that lung cancer in the young is enriched for targetable genomic alterations. compared with older patients (5, 6).

Despite the efforts to characterize the genomic profile of NSCLC in the young, the information on this subset of patients remains limited, especially in Latin American countries which are underrepresented in international genomic databases. The purpose of this study is to evaluate the genomic profile of young NSCLC patients and to compare it with the profile of older patients.

## Methods

### Study design and population

We included NSCLC patients aged ≤40 years old diagnosed at Instituto Nacional de Enfermedades Neoplásicas (INEN) between 2009-2018 as cases and NSCLC patients aged >40 years old diagnosed between 2013-2015, as controls. Archival tissue with cellularity over 20% suitable for next generation sequencing (NGS) was mandatory for inclusion, as well as available clinical data. The sample collection period is longer for cases due to the scarcity of NSCLC patients aged ≤40 years with suitable samples.

### Clinicopathological and genomic variables

Variables analyzed in both age groups included sex, smoking status, histology, and clinical stage. For the genomic analysis, we included pathogenic and likely pathogenic alterations as reported in COSMIC (7). A targetable alteration was defined as any alteration in a driver oncogene for which an FDA approved therapy existed at the time of study enrollment.

### Next generation sequencing

The genomic analysis was conducted in formalin-fixed and paraffin embedded (FFPE) tumors. Samples with at least 20% of tumor cellularity, as assessed by an expert pathologist, were submitted to Foundation Medicine. The genomic analysis was performed with targeted NGS using the platform FoundationOne CDx^®^ (Foundation Medicine, Cambridge, USA). This test identifies short nucleotide changes, insertions, deletions, copy number variations, gene tumor mutational burden (TMB), and microsatellite instability (MSI) in 324 driver genes and rearrangements in 28 commonly rearranged genes in malignant tumors. TMB is defined by the FoundationOne CDx^®^ assay as the total number of all synonymous and nonsynonymous variants present at >5% allele frequency and reported as mutations per megabase (mut/Mb) unit. TMB is classified in low (≤5 mut/Mb), intermediate (6 to 19 Mut/Mb), and high TMB (≥20Mut/Mb).

### Statistical analysis

Statistical analysis was conducted using RStudio version 1.2.5033 based on the statistical language R version 3.6.3. The Fisher’s exact test was used to compare the percentage of shared alterations in the tumor samples from younger versus older patients. The Wilcoxon test was used for comparison of TMB. Genomic alterations were plotted using the GenVisR and the ggplot2 package (8). Due to the exploratory nature of this study, P-values were not adjusted for multiple hypothesis testing.

### Ethical considerations

This study was approved by the Institutional Review Board of INEN.

## Results

### Characteristics of patients

**Figure 1:**
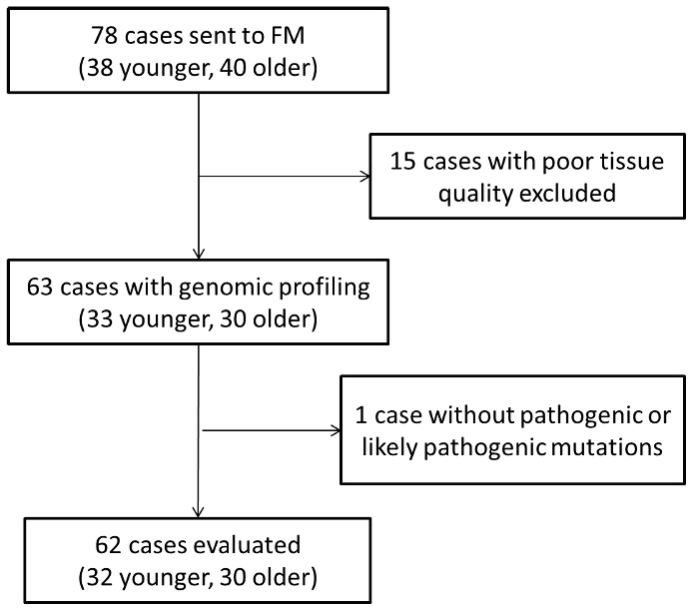
Flowchart of the patient included in the study. FM, Foundation Medicine.

Overall, 38 younger patients and 40 older patients met the inclusion criteria, and their samples were sent to Foundation Medicine for profiling. Fifteen samples were excluded due to poor quality of the tissue, or the DNA extracted. One patient was excluded from the statistical analysis due to the absence of pathogenic or likely pathogenic mutations. Finally, 32 younger and 30 older patients were included for final analysis (**Figure 1**). In 3 younger and in 2 older patients it was not possible to estimate the TMB.

**Figure 2:**
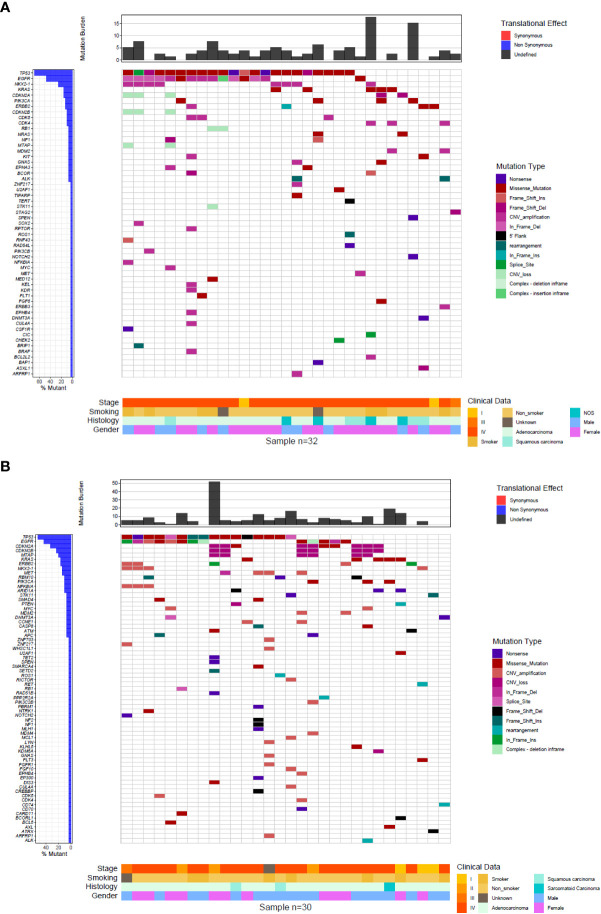
Comprehensive visualization of the genomic landscape of NSCLC. **(A)** young **(B)** older patients.

The median age for young patients was 39.7 years old (DE ± 6.8) and for the older ones 62.2 years (DE ± 8.9). There was a similar distribution of clinicopathological variables (smoking status, histology, and clinical stage) between the age groups except for sex, being female predominant among younger patients (65.6% vs 40%, P=0,043) (**Table 1**). Correlation between mutations and clinicopathological features is shown in **Figure 2**.

**Table 1:** Comparison in clinical characteristics between younger and older patients with NSCLC.

Characteristics	Younger		Older		P- value*
	n	%	n	%	
**Sex**					**0.043**
Female	21	65.6	12	40.0	
Male	11	34.1	18	60.0	
**Smoking status**					0.942
Non Smoker	23	76.7	22	76.3	
Smoker	7	23.3	7	23.7	
Unknown	2		1		
**Histology**					0.208**
Adenocarcinoma	23		27		
Squamous cell carcinoma	5		2		
Sarcomatoid carcinoma	0		1		
NOS	4		0		
**Clinical stage**					0.12
I-III	3	9.4	7	25.1	
IV	29	90.6	22	75.9	

*, Chi-square test; **, comparison between adenocarcinoma vs. squamous cell carcinoma. P-values statistically significant.

### Comparison of short-variants between younger or older patients with NSCLC

**Figure 3:**
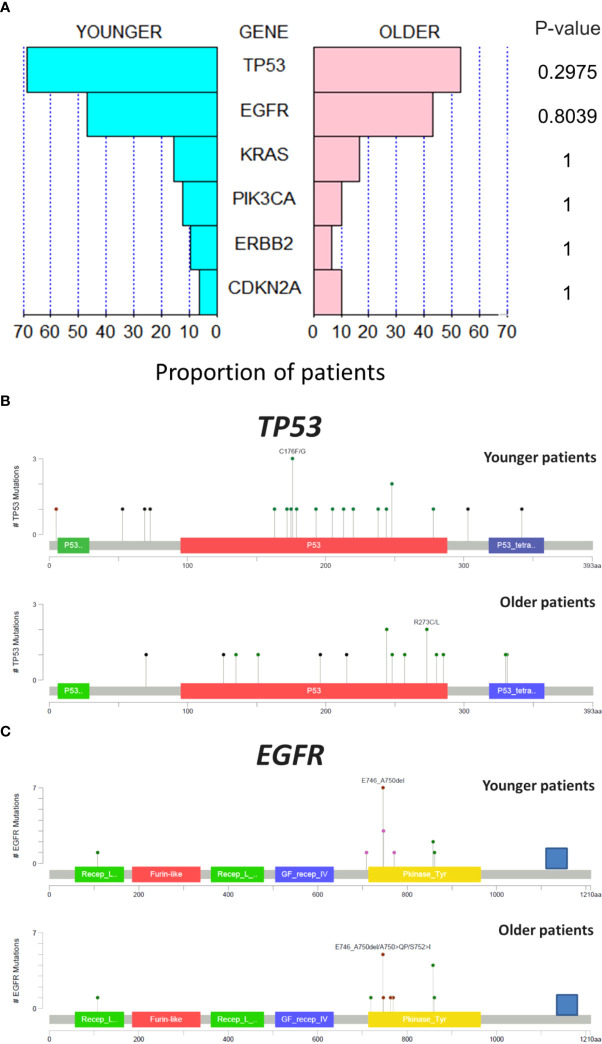
Comparative analysis of mutation between age groups in genes more frequently altered **(A)**. Comparison between age groups of the structural alteration in p53 **(B)** and in EGFR **(C)**.

In total, 78 vs 89 genes with actionable mutations were detected in younger vs older patients with NCSLC, respectively (**Figure 2**). There were statistically significant differences between the number of co-mutated genes between younger vs older patients (means of 2.5 vs 3 co-mutations per patient, respectively; P<0.001). *TP53* mutations were present in 68.8% of cases (n=22) of NSCLC younger patients and in 53.3% (n=16) of NSCLC older patients (P=0.2975). *EGFR* mutations were observed in 46.9% of tumor samples of younger (n=15) and in 43.3% of samples of older patients with NSCLC (n=13) (P= 0.8039). 15.6% of younger (n=5) and 16.7% (n=5) of younger patients had alterations in *KRAS* (p=0.9113). KRAS alterations were found in young were G12V (n=2), G12D (n=2), G12C (n=1); while in older patients, KRAS alterations include G12V (n=1), G12D (n=3), Q61L(n=1). Mutations in PIK3CA were present in 12.5% of younger (n=4) and 10% of older patients (n=3) (P=1.0). *ERBB2* alterations were present in 9.4% (n=3) of younger and 6.7% (n=2) of older patients (P=1.0) and 6.3% (n=2) and 10% (n=3) had alterations in CDKN2A (p=0.5879), respectively (**Figure 3A**).

The mutation regions for *TP53* and *EGFR*, the most altered genes, were explored. For p53 gene, tumors from younger patients presented more mutations out of the DNA-binding domain than tumors from older patients (**Figure 3B**). For *EGFR* gene, a similar pattern in the position of the alterations was observed with most of the mutations located at the tyrosine kinase domain (**Figure 3C**).

### Comparative analysis of TMB

**Figure 4:**
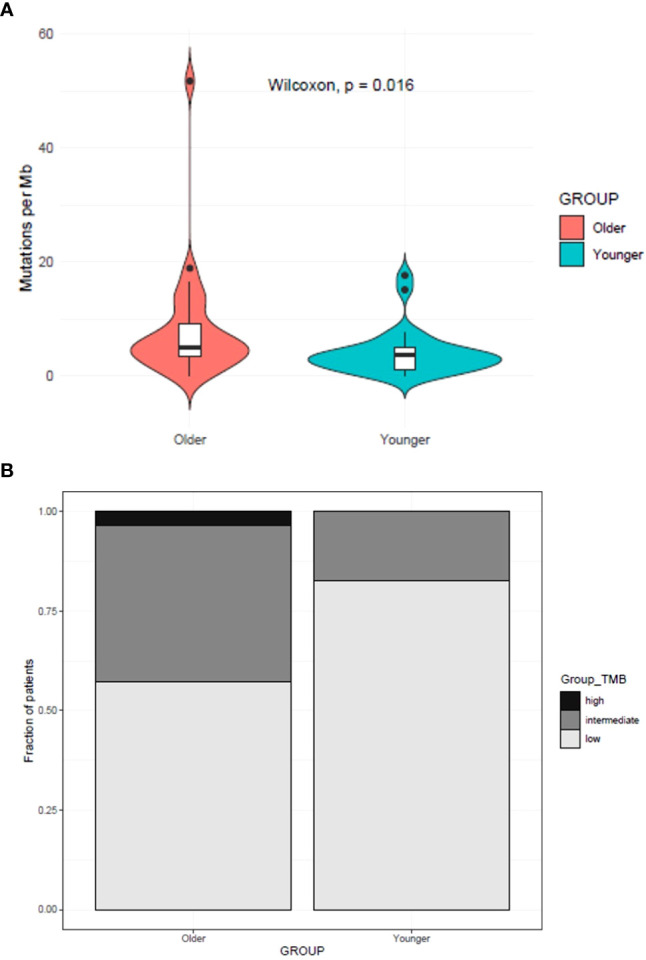
TMB comparison between younger and older patients with NSCLC **(A)**. **(B)** Distribution of TMB groups between younger with older patients **(B)**.

The TMB count for each case is shown in **Figure 2**. The mean TMB for tumors from younger patients was 4.04 Mut/Mb (SD ± 3.98) vs. 8.06 Mut/Mb (SD ± 9.84) for older patients, with a statistically significant difference (P=0.016) (**Figure 4A**). Regarding the distribution of TMB groups, 82.8% of young patients (n=24) had low TMB and 17.2% (n=5) had an intermediate TMB, while 57.1% of older patients (n=16) had low TMB, 39.3% (n=11), had intermediate TMB and one patient (3.6%) had a high TMB (P=0.05614) (**Figure 4B**). To further explore if EGFR status could explain the difference, TMB in EGFR mutated vs wild type was assessed (regardless age group) showing no significant differences (**Figure S1**).

### Comparison of CNVs, rearrangements and microsatellite instability

**Figure 5:**
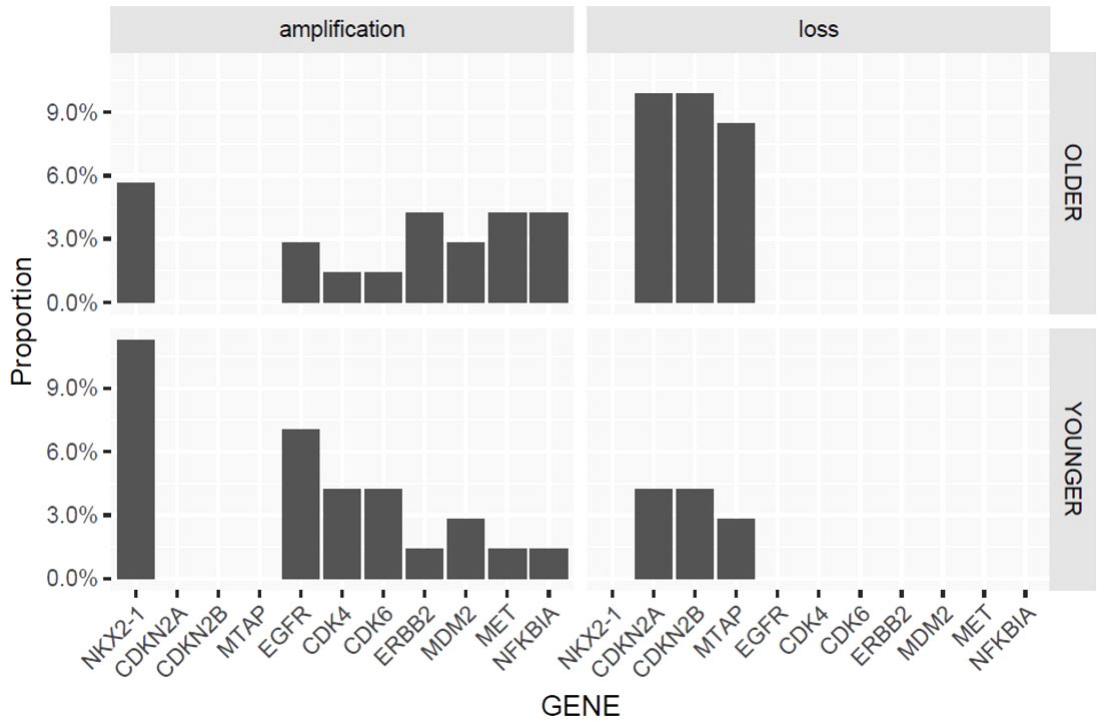
Copy number variation in older vs younger patients.

CNVs were detected in 42 genes in both groups. Genes with higher frequencies of CNVs included CDK4, CDK6, CDKN2A, CDKN2B, EGFR, ERBB2, MDM2, MET, MTAP, NFKBIA, NFKBIA and NKX2-1. There was not a significant difference between age groups in the distribution of genes with amplification or loss (**Figure 5**).

Four gene rearrangements were detected in younger patients and 5 in older patients. Rearranged genes in tumors from younger patients included ROS1, ALK, and BRIP1, while genes with this type of alteration in older patients were ROS1, PPP2R2A, CD74, PTEN, RET and ALK (**Table 2**).

**Table 2:** Comparison of rearrangements detected in younger vs older patients with NSCLC.

GROUP	GENE 1	GENE 2	n	DESCRIPTION
YOUNGER PATIENTS	ROS1	EZR	1	fusion
	ALK	EML4	2	fusion
	BRIP1	MYST3	1	truncation
OLDER PATIENTS	ROS1	N/A	1	rearrangement
	PPP2R2A	BNIP3L	1	truncation
	CD74	NRG1	1	fusion
	PTEN	AAGAB	1	truncation
	RET	KIF5B	1	fusion
	ALK	EML4	1	fusion

Microsatellite instability (MSI-H) was only detected in one case of a younger patient.

### Comparison of actionable mutations (BRAF, EGFR, ERBB2, KRAS, ALK, RET, ROS1, NTRK, MET)

The rate of any actionable mutation was compared between young vs. older patients, including mutations in short variants, gene amplifications, and gene rearrangements. In total, 27 out 32 (84.4%) young patients had at least one targetable mutation vs. 25 out 30 older patients (83.3%). The alteration rates were as follows: BRAF, 3.1%(n=1) vs 0%; EGFR, 46.9% (n=15) vs 43.3% (n=13); ERBB2, 12.5% (n=4) vs 16.7% (n=5); KRAS, 15.6% (n=5) vs 16.7% (n=5); ALK, 6.3% (n=2) vs 3.3% (n=1); RET, 0.0% vs 3.3% (n=1); ROS1, 3.1% (n=1) vs 3.3% (n=1); NTRK1, 0.0% vs 3.3% (n=1) and MET, 3.1% (n=1) vs 13.3% (n=4).

## Discussion

Lung cancer in the young is uncommon, accounting for 3% of all cases worldwide (9). This entity is associated with a more aggressive biology and worse prognosis, although also present a high frequency of targetable mutations (10). Although there are an increasing number of publications about genomic alterations in NSCLC in young patients (10–12), this is, to our knowledge, the first comprehensive analysis of lung cancer in the young. Previous studies in this population have evaluated small gene panels with limited information about other molecular features than point mutations or selected rearrangements.

The etiology of lung cancer in young patients is unclear. A great proportion of lung cancer patients <40 years are women and never-smokers (13). Interestingly, a preliminary report by Gitlitz et al. (2017), proposes a relationship between several environmental risk factors (including passive smoking) with specific genomic alterations. On the other hand, only two single polymorphism nucleotides were associated with the risk of lung cancer (11). An important set of molecular alterations seen in young patients could correspond to germline mutations. Donner et al. (14), found that germline mutations in BRCA1, BRCA2, ERCC4, EXT1, HNF1A, PTCH1, SMARCB1 and TP53 were related with the development of lung adenocarcinoma in never smoker young women (14). Further studies will provide more information about the etiology of lung cancer in young patients.

Various studies have reported a high frequency of actionable mutations in young patients with NSCLC. In a study of 2237 patients treated at the Dana Farber Cancer Institute, among young patients (<40 years), 32% exhibited alterations in EGFR and 19% in ALK. Moreover, patients diagnosed with NSCLC at a younger age had an increased likelihood of EGFR kinase mutations (P = .02) and ALK rearrangements (P <.001) (10). Likewise, Pan et al. (15), in a study which included 252 Chinese patients aged ≤40 years, reported 40%, 34%, and 14% of alterations in EGFR, ALK and ROS1, respectively (15). While EGFR alterations fall into what it is expected for this population, ALK and ROS1 alteration rates were remarkably high. Likewise, a study involving 7858 lung cancer patients which compared mutations between patients aged ≤45 vs >45 years observed a higher prevalence of ALK, ROS1 and RET fusions, ERBB2 exon-20 insertions and EGFR exon-19 deletions in younger patients (16). The enrichment of actionable mutation in young patients has been reported also in a retrospective cohort from Israel (17).

Regarding TP53, its relatively high incidence is comparable to that reported for Latin American countries (18, 19). This incidence, as well as the high frequency of other mutations reported in this study, particularly EGFR could be associated with a particular genetic ancestry (20). In fact, according to data from the Cancer Genome Atlas Database, EGFR mutated patients exhibit a higher rate of mutated type TP53 than EGFR wild type patients (21).

In Latin America, CLICaP assessed EGFR and ALK status in 389 patients under 40 years old and found that EGFR mutations and EML4-ALK fusion were present in 70.8% and 10.1% of cases, respectively (12). In this case, the rate of EGFR mutation is exceedingly high, and a selection bias could have influenced the results. It is important to note that no comparison group was considered for this study.

On the contrary, in the present study, we did not find significant differences in the proportion of targetable mutations between age groups. Neither did we find any significant differences in the rate of EGFR (46.5% vs 43.3%). It appears that EGFR mutations, which are very frequent in our population and cause most of the difference in the proportion of targetable mutations between age groups in other studies, occur at the same rate in Peruvian patients with NSCLC younger and older. This is consistent with a recent Chinese report which included 1472 patients and found that EGFR mutation rates were not significantly different between younger (≤45 years) and older patients (52.6% vs 52.0%) (22). ALK rearrangements, which have been consistently reported as being more frequent in the young (5, 6, 15), were not significantly different in our cohort.

Herein, unlike prior experiences, a similar distribution of clinicopathological variables between the age groups was found except for sex with a female preponderance among the young. To some extent, the molecular differences between young vs older patients described in other reports and not encountered in the present study, might be attributed to differences in the distribution of some clinicopathological characteristics. For example, in the Dana Farber cohort, when examining individual mutations within a multivariate model correcting for smoking status, female sex, and Asian race; the association between EGFR mutations and age was no longer significant and the only genotype associated with a younger age at diagnosis was ALK rearrangement (10). Also, in Peru lung cancer occurs predominantly in never-smokers (23), which may not be true for other settings in which lung cancer occurs predominantly in smokers with young lung cancer patients representing an etiologically different group (never-smokers). Having said this, due to the small sample size in our study, some statistical differences in genomic alterations could be hid because of the sample size, leading to a high probability of type-2 error. Further evaluations and a well-powered sample size are needed to confirm this hypothesis. In this sense, collaboration with other Peruvian institutions is ongoing.

It has been consistently reported that TMB is positively associated with TP53 alterations, smoking, squamous cell histology, and male sex; and negatively associated with EGFR mutations (6, 24). TMB is typically low in oncogene-addicted tumors, which represent an important proportion of our cohort. In our study, TMB was overall low in both age groups, as well as it was the smoking prevalence and the squamous cell subtype. Moreover, TMB was significantly lower in the younger age group when compared with the older one, regardless of the presence of EGFR mutation. The age itself and the higher proportion of female patients in the younger group could explain this difference. Importantly, TMB influences prognosis in patients whose tumors harbor driver mutations; a lower TMB has been associated with an improved benefit from EGFR TKI and on the contrary, high TMB may translate an increased prevalence of resistance mechanisms and subclones (25). It has also been shown that patients with low TMB have a worse response to immunotherapy when compared with patients with high TMB (6, 26).

Our study has some weaknesses. First, 19% of our samples were not suitable for genomic analysis. Also, due to the scarcity of NSCLC patients aged ≤40 years with suitable samples the group of younger patients included was diagnosed in a 10-year period while the group of older patients was diagnosed more recently. Because of sample size and the lack of a contemporary comparator group the intention of this study was not to evaluate clinical outcomes or prognosis, therefore the interpretation bias diminishes. Moreover, it was only by 2017 that access to targeted therapy in the subsidized regimen was granted (23), therefore practically none of the patients had the opportunity to receive this treatment.

In conclusion, lung cancer in the young in our cohort was characterized by a high frequency of actionable genetic aberrations and a low TMB. This is also true for our older patients, leading to a great opportunity of treatment with targeted therapy for our whole population. The differences in the genomic landscape and the enrichment of actionable mutations in young patients previously reported by multiple series might be attributed to differences in etiology and clinicopathological characteristics between younger and older patients and therefore not be applicable to all populations.

## Data availability statement

The original contributions presented in the study are included in the article/**Supplementary Material**. Further inquiries can be directed to the corresponding author.

## Ethics statement

The studies involving human participants were reviewed and approved by IRB of Instituto Nacional de Enfermedades Neoplásicas. Written informed consent for participation was not required for this study in accordance with the national legislation and the institutional requirements.

## Author contributions

Conception and design: RR, SS-G, LM, JP Acquisition of data: RR, MG-N, KR, JM, MN. Assembly of data: RR, JP Bioinformatic analysis: JP Analysis and interpretation of data: RR, MG-N, KR, JM, MN, LR, LM, JP. Writing, review, and/or revision of the manuscript: All authors Final approval of the manuscript: All authors.

## Funding

The authors declare that this study received funding from Roche Peru. The funder was not involved in the study design, collection, analysis, interpretation of data, the writing of this article or the decision to submit it for publication.

## Conflict of interest

LR has received grants for research support to Institution from Roche, Pfizer, Genetech, BMS, Lilly, Novartis, Syndax, Liquid Genomics and Astra Zeneca. JAP has received payments for scientific projects development from Roche Peru and a research and travel grant from Foundation Medicine. SJ, SV and ES are employees of Roche Farma, Peru. SSG worked at Roche Peru as RWData/Evidence manager Until July 2021.

The remaining authors declare that the research was conducted in the absence of any commercial or financial relationships that could be construed as a potential conflict of interest.

## Publisher’s note

All claims expressed in this article are solely those of the authors and do not necessarily represent those of their affiliated organizations, or those of the publisher, the editors and the reviewers. Any product that may be evaluated in this article, or claim that may be made by its manufacturer, is not guaranteed or endorsed by the publisher.

## Supplementary material

Supplementary Table 1 Parameters of SV Coverage and SV percent of read.

Supplementary Table 2 Samples evaluated for TMB.
