# Supplementary material for: Genomic landscape of lung cancer in the young
Source: Front Oncol. 2022 Sep 29;12:910117. doi: 10.3389/fonc.2022.910117 (PMC9575317; doi:10.3389/fonc.2022.910117)
Supplement: Supplementary Table 1 — Parameters of SV Coverage and SV percent of read. [file DataSheet_1.pdf]

Specimen Name	QC Status	Disease	Gender
ORD-0741907-01	PASS	unknown primary adenocarcinoma	M
ORD-0741909-01	QUALIFIED	unknown primary adenocarcinoma	F
ORD-0741910-01	PASS	unknown primary adenocarcinoma	F
ORD-0741912-01	PASS	unknown primary adenocarcinoma	F
ORD-0741913-01	PASS	unknown primary adenocarcinoma	M
ORD-0741915-01	PASS	unknown primary sarcomatoid carcinoma	M
ORD-0741916-01	PASS	unknown primary squamous cell carcinoma (sc	M
ORD-0741917-01	PASS	unknown primary adenocarcinoma	M
ORD-0741920-01	PASS	unknown primary adenocarcinoma	M
ORD-0741921-01	PASS	unknown primary squamous cell carcinoma (sc	M
ORD-0741922-01	PASS	unknown primary adenocarcinoma	M
ORD-0741925-01	QUALIFIED	unknown primary adenocarcinoma	F
ORD-0741926-01	QUALIFIED	unknown primary adenocarcinoma	M
ORD-0741927-01	QUALIFIED	unknown primary adenocarcinoma	M
ORD-0741928-01	PASS	unknown primary adenocarcinoma	F
ORD-0741929-01	PASS	unknown primary adenocarcinoma	F
ORD-0741930-01	PASS	lung adenocarcinoma	M
ORD-0741932-01	QUALIFIED	unknown primary adenocarcinoma	F
ORD-0741933-01	PASS	unknown primary adenocarcinoma	F
ORD-0741934-01	PASS	unknown primary adenocarcinoma	F
ORD-0741935-01	PASS	unknown primary adenocarcinoma	M
ORD-0741936-01	QUALIFIED	unknown primary adenocarcinoma	M
ORD-0741937-01	PASS	unknown primary adenocarcinoma	F
ORD-0741938-01	PASS	unknown primary adenocarcinoma	M
ORD-0741939-01	PASS	unknown primary adenocarcinoma	M
ORD-0741940-01	PASS	unknown primary adenocarcinoma	M
ORD-0741941-01	PASS	unknown primary adenocarcinoma	F
ORD-0741942-01	PASS	unknown primary adenocarcinoma	M
ORD-0741944-01	QUALIFIED	unknown primary adenocarcinoma	F
ORD-0741945-01	PASS	unknown primary adenocarcinoma	F
ORD-0741946-01	PASS	unknown primary carcinoma (nos)	F
ORD-0741947-01	QUALIFIED	unknown primary adenocarcinoma	M
ORD-0741948-01	QUALIFIED	unknown primary carcinoma (nos)	M
ORD-0741949-01	QUALIFIED	unknown primary squamous cell carcinoma (sc	F
ORD-0741951-01	PASS	lung adenocarcinoma	M
ORD-0741952-01	QUALIFIED	unknown primary adenocarcinoma	F
ORD-0741953-01	QUALIFIED	unknown primary squamous cell carcinoma (sc	F
ORD-0741954-01	PASS	unknown primary adenocarcinoma	F
ORD-0741955-01	QUALIFIED	unknown primary adenocarcinoma	F
ORD-0741959-01	QUALIFIED	lung adenocarcinoma	F
ORD-0741960-01	QUALIFIED	unknown primary carcinoma (nos)	M
ORD-0741961-01	PASS	unknown primary squamous cell carcinoma (sc	F
ORD-0741962-01	QUALIFIED	lung adenocarcinoma	F
ORD-0741963-01	QUALIFIED	lung adenocarcinoma	F
ORD-0741965-01	PASS	unknown primary adenocarcinoma	M

ORD-0741967-01	QUALIFIED	lung adenocarcinoma	F
ORD-0741969-01	PASS	unknown primary adenocarcinoma	F
ORD-0741970-01	PASS	unknown primary adenocarcinoma	M
ORD-0741971-01	PASS	unknown primary adenocarcinoma	F
ORD-0741972-01	PASS	unknown primary adenocarcinoma	F
ORD-0741973-01	PASS	unknown primary adenocarcinoma	F
ORD-0741974-01	PASS	unknown primary adenocarcinoma	M
ORD-0741975-01	QUALIFIED	unknown primary adenocarcinoma	F
ORD-0741976-01	PASS	unknown primary adenocarcinoma	F
ORD-0741977-01	PASS	lung squamous cell carcinoma (scc)	M
ORD-0741978-01	PASS	unknown primary adenocarcinoma	F
ORD-0741979-01	PASS	unknown primary adenocarcinoma	M
ORD-0741980-01	PASS	unknown primary adenocarcinoma	M
ORD-0741981-01	PASS	unknown primary squamous cell carcinoma (scc	M
ORD-0741982-01	PASS	unknown primary carcinoma (nos)	F
ORD-0741983-01	PASS	unknown primary adenocarcinoma	F
ORD-0741984-01	PASS	unknown primary adenocarcinoma	F
ORD-0742170-01	PASS	unknown primary adenocarcinoma	M

######FOR RESEARCH USE ONLY######

CLIA TMB (mutations per megabase)	CLIA TMB Level
6.3	intermediate
5.04	low
5.04	low
6.3	intermediate
16.39	intermediate
18.91	intermediate
3.78	low
2.52	low
12.61	intermediate
7.57	intermediate
1.26	low
7.57	intermediate
3.78	low
unknown	unknown
5.04	low
0	low
0	low
13.87	intermediate
13.87	intermediate
3.78	low
2.52	low
unknown	unknown
1.26	low
5.04	low
2.52	low
10.09	intermediate
8.83	intermediate
5.04	low
5.04	low
51.69	high
6.3	intermediate
2.52	low
unknown	unknown
1.26	low
2.52	low
unknown	unknown
5.04	low
2.52	low
3.78	low
0	low
3.78	low
15.13	intermediate
2.52	low
unknown	unknown
1.26	low

1.26	low
3.78	low
1.26	low
2.52	low
7.57	intermediate
3.78	low
3.78	low
1.26	low
5.04	low
1.26	low
2.52	low
0	low
5.04	low
0	low
17.65	intermediate
3.78	low
7.57	intermediate
3.78	low
