# Supplementary material for: Genomic landscape of lung cancer in the young
Source: Front Oncol. 2022 Sep 29;12:910117. doi: 10.3389/fonc.2022.910117 (PMC9575317; doi:10.3389/fonc.2022.910117)
Supplement: Supplementary Table 2 — Samples evaluated for TMB. [file DataSheet_2.pdf]

SAMPLE ID	VARIANT-TYI	GENE	SOMATIC STATUS/FUI	SV-PROTEIN-CHAN	SV-CDS-CHANGE
279997	short-variant	CD70	likely	E51*	151G>T
279997	short-variant	EGFR	known	L858R	2573T>G
279997	copy-number	CDK4	known	-	-
279997	copy-number	CCNE1	known	-	-
279997	copy-number	CDKN2A	known	-	-
279997	copy-number	MDM2	known	-	-
279997	copy-number	MTAP	known	-	-
279997	copy-number	EPHB4	known	-	-
279997	copy-number	CDKN2B	known	-	-
279997	copy-number	MET	known	-	-
304330	short-variant	MET	likely	D972_D1002del	2914_3006del93
304330	short-variant	TP53	known	C135Y	404G>A
304330	copy-number	CDKN2B	known	-	-
304330	copy-number	CDKN2A	known	-	-
304330	copy-number	MTAP	known	-	-
297613	short-variant	EGFR	known	L861Q	2582T>A
297613	short-variant	EGFR	known	G719S	2155G>A
297613	copy-number	ERBB2	known	-	-
297613	copy-number	MDM2	known	-	-
283515	short-variant	CDKN2A	known	H83Y	247C>T
283515	short-variant	EGFR	known	E746_A750del	2235_2249delGGA
286922	short-variant	TP53	likely	splice site 920-1G	920-1G>T
286922	short-variant	STK11	known	Q220*	658C>T
286922	copy-number	MCL1	known	-	-
286922	copy-number	CUL4A	known	-	-
286922	copy-number	RICTOR	known	-	-
286922	copy-number	FGF10	known	-	-
278738	short-variant	PIK3CA	known	E545K	1633G>A
278738	short-variant	AXL	known	R368Q	1103G>A
278738	short-variant	KRAS	known	G12V	35G>T
278738	copy-number	KRAS	known	-	-
277242	short-variant	CDKN2A	known	A85P	253G>C
277242	short-variant	ARID1A	likely	S2042fs*93	6123delG
277242	short-variant	TP53	known	L257Q	770T>A
277242	copy-number	PTEN	known	-	-
310798	short-variant	EGFR	known	L858R	2573T>G
310798	short-variant	APC	likely	L1342fs*10	4023_4024insAACC
310798	short-variant	EGFR	known	R108K	323G>A
310798	short-variant	SMAD4	known	D493N	1477G>A
310798	short-variant	TP53	known	P151T	451C>A
310798	copy-number	CDK6	known	-	-
314699	short-variant	EP300	likely	L84*	251T>A
314699	short-variant	CREBBP	likely	L524fs*6	1570delC
314699	short-variant	SMARCA4	known	T910M	2729C>T
314699	short-variant	PBRM1	known	R1027*	3079C>T
314699	short-variant	NF2	likely	Q165fs*37	494_495delAA
314699	short-variant	TP53	known	Q331R	992A>G

314699	short-variant	TP53	known	L330R	989T>G
314699	short-variant	NF1	likely	R1970fs*6	5907_5908delAA
314699	short-variant	SMAD4	known	R361C	1081C>T
314699	short-variant	CASP8	likely	L239fs*37	716_717insT
314699	short-variant	MLH1	likely	G232*	694G>T
314699	copy-number	MET	known	-	-
330959	short-variant	RBM10	likely	Q601*	1801C>T
330959	short-variant	TP53	known	R280K	839G>A
330959	rearrangeme	ROS1	likely	-	-
318068	short-variant	KRAS	known	G12D	35G>A
318068	short-variant	ARID1A	likely	Q553*	1657C>T
318068	copy-number	CDKN2A	known	-	-
318068	copy-number	CDKN2B	known	-	-
318068	copy-number	KDM6A	known	-	-
317791	short-variant	EGFR	known	L858R	2573T>G
317791	short-variant	CDKN2A	known	P81L	242C>T
317791	rearrangeme	PPP2R2A	likely	-	-
332380	short-variant	TP53	likely	S215fs*33	643_644insTA
332380	short-variant	EGFR	known	V769_D770insASV	2308_2309insCCAG
1.7E+09	short-variant	DNMT3A	likely	Q573*	1717C>T
1.7E+09	rearrangeme	CD74	known	-	-
1.6E+09	short-variant	TP53	likely	A70fs*52	207_210delTGCT
1.6E+09	short-variant	KRAS	known	G12D	35G>A
1.6E+09	copy-number	CCNE1	known	-	-
1.6E+09	short-variant	ATRX	likely	L1854fs*1	5561delT
1.6E+09	short-variant	STK11	likely	E130fs*33	388_389insG
1.6E+09	short-variant	BCORL1	likely	V1176fs*42	3526_3538delGTCA
1.6E+09	short-variant	KRAS	known	G12D	35G>A
1.6E+09	short-variant	U2AF1	known	S34F	101C>T
1.6E+09	short-variant	ARID1A	known	Q633*	1897C>T
1.6E+09	rearrangeme	PTEN	likely	-	-
1.6E+09	short-variant	TP53	known	R273C	817C>T
1.6E+09	short-variant	CARD11	known	R271W	811C>T
1.6E+09	short-variant	EGFR	known	L858R	2573T>G
1.6E+09	short-variant	RB1	likely	splice site 1390-22	1390-226_1422-29
1.6E+09	short-variant	FLT3	known	M664I	1992G>C
1.6E+09	copy-number	NKX2-1	known	-	-
1.6E+09	rearrangeme	RET	known	-	-
1.6E+09	short-variant	KRAS	known	Q61L	182A>T
1.6E+09	short-variant	RBM10	likely	R6fs*128	17delG
1.6E+09	short-variant	KLHL6	known	T274M	821C>T
1.6E+09	copy-number	MTAP	known	-	-
1.6E+09	copy-number	CDKN2B	known	-	-
1.6E+09	copy-number	CDKN2A	known	-	-
1.5E+09	short-variant	TP53	likely	Y126fs*26	370_371insGCACG
1.5E+09	short-variant	EGFR	known	E746_S752>I	2236_2255GAATTA
1.5E+09	short-variant	DNMT3A	likely	splice site 2478+2T	2478+2T>G
1.5E+09	short-variant	EGFR	known	L747_S752del	2239_2256delTTAA

1.5E+09	short-variant	BCL6	known	N73S	218A>G
1.5E+09	short-variant	TP53	likely	splice site 559+1G:	559+1G>A
1.5E+09	copy-number	MYC	known	-	-
1.5E+09	copy-number	EGFR	known	-	-
1.5E+09	short-variant	EGFR	known	A763_Y764insFQE/	2290_2291insTCCA(
1.5E+09	short-variant	NOTCH2	known	Q1392*	4174C>T
1.5E+09	short-variant	TP53	known	R273L	818G>T
1.5E+09	copy-number	ZNF217	known	-	-
1.5E+09	copy-number	NFKBIA	known	-	-
1.5E+09	copy-number	ERBB2	known	-	-
1.5E+09	copy-number	NKX2-1	known	-	-
1.6E+09	short-variant	EGFR	known	E746_A750>QP	2236_2248GAATTA
1.6E+09	short-variant	APC	known	R216*	646C>T
1.6E+09	short-variant	PIK3CA	known	E545A	1634A>C
1.6E+09	copy-number	MTAP	known	-	-
1.6E+09	copy-number	CDKN2B	known	-	-
1.6E+09	copy-number	CDKN2A	known	-	-
1.6E+09	copy-number	MDM4	known	-	-
1.6E+09	copy-number	PIK3C2B	known	-	-
1.6E+09	short-variant	PIK3CA	known	M1004I	3012G>A
1.6E+09	short-variant	CASP8	known	E179D	537G>C
1.6E+09	copy-number	CDKN2A	known	-	-
1.6E+09	copy-number	MTAP	known	-	-
1.6E+09	copy-number	CDKN2B	known	-	-
1.6E+09	copy-number	MYC	known	-	-
1.6E+09	rearrangeme	ALK	known	-	-
1.5E+09	short-variant	TP53	known	G244S	730G>A
1.5E+09	short-variant	RBM10	likely	T371fs*10	1111_1112insA
1.5E+09	short-variant	NTRK1	known	R508W	1522C>T
1.5E+09	short-variant	EGFR	known	E746_A750del	2235_2249delGGA/
1.5E+09	copy-number	EGFR	known	-	-
1.5E+09	copy-number	NFKBIA	known	-	-
1.5E+09	copy-number	NKX2-1	known	-	-
1.5E+09	short-variant	TP53	known	R248L	743G>T
1.5E+09	copy-number	WHSC1L1	known	-	-
1.5E+09	copy-number	ARFRP1	known	-	-
1.5E+09	copy-number	FGFR1	known	-	-
1.5E+09	copy-number	MET	known	-	-
1.5E+09	copy-number	LYN	known	-	-
1.5E+09	copy-number	GNAS	known	-	-
1.5E+09	copy-number	ZNF703	known	-	-
1.5E+09	short-variant	TP53	known	G244D	731G>A
1.5E+09	short-variant	EGFR	known	E746_A750del	2236_2250delGAAT
1.5E+09	short-variant	TP53	known	R196*	586C>T
1.5E+09	copy-number	ERBB2	known	-	-
1.5E+09	copy-number	NKX2-1	known	-	-
1.5E+09	copy-number	NFKBIA	known	-	-
1.8E+09	short-variant	DIS3	known	D458N	1372G>A

1.8E+09	short-variant	TET2	likely	E1755*	5263G>T
1.8E+09	short-variant	SETD2	likely	K637fs*1	1908_1909insT
1.8E+09	short-variant	TP53	known	E285K	853G>A
1.8E+09	short-variant	RAD51B	likely	E198*	592G>T
1.8E+09	short-variant	ATM	known	R2443Q	7328G>A
1.8E+09	short-variant	SPEN	likely	E2176*	6526G>T
1.8E+09	short-variant	ERBB2	known	G776>VC	2326_2327insTGT
1.8E+09	copy-number	CDKN2A	known	-	-
1.8E+09	copy-number	CDKN2B	known	-	-
1.8E+09	copy-number	MTAP	known	-	-
201722	short-variant	BAP1	likely	Q483*	1447C>T
201722	short-variant	NRAS	known	Q61R	182A>G
201722	short-variant	TP53	known	C176F	527G>T
201722	short-variant	NF1	likely	K2664fs*1	7989_7990insT
201722	short-variant	PIK3CA	known	E545K	1633G>A
202039	short-variant	STAG2	likely	P5fs*66	14delC
217733	short-variant	CDKN2A	likely	E120fs*26	358delG
217733	short-variant	NRAS	known	Q61R	182A>G
228390	short-variant	EGFR	known	L858R	2573T>G
228390	copy-number	MET	known	-	-
235619	short-variant	EGFR	known	E746_A750del	2235_2249delGGA/
235619	short-variant	TP53	known	Y163C	488A>G
235619	copy-number	NKX2-1	known	-	-
272879	short-variant	TP53	known	Y205C	614A>G
272879	short-variant	EGFR	known	R108K	323G>A
272879	short-variant	EGFR	known	L861Q	2582T>A
272879	short-variant	PIK3CA	known	E545K	1633G>A
277550	short-variant	RAD54L	likely	Q635*	1903C>T
277550	short-variant	TERT	known	promoter -124C>T	-124C>T
277550	short-variant	TP53	known	P278L	833C>T
277550	rearrangeme	ROS1	known	-	-
281531	short-variant	KRAS	known	G12D	35G>A
281531	copy-number	MDM2	known	-	-
281531	copy-number	CDK4	known	-	-
1.6E+09	short-variant	TP53	likely	V73fs*50	210delT
1.6E+09	short-variant	EGFR	known	E709_T710>D	2127_2129delAAC
1.6E+09	copy-number	PIK3CB	known	-	-
1.6E+09	copy-number	NKX2-1	known	-	-
1.6E+09	short-variant	ERBB2	known	A775_G776insYVM	2324_2325insATAC
1.6E+09	short-variant	TP53	known	C238G	712T>G
1.6E+09	copy-number	NKX2-1	known	-	-
1.6E+09	short-variant	PIK3CA	known	E545K	1633G>A
1.6E+09	short-variant	NOTCH2	likely	Q211*	631C>T
1.6E+09	short-variant	SPEN	likely	R882*	2644C>T
1.6E+09	short-variant	EGFR	known	E746_A750del	2236_2250delGAAT
1.6E+09	short-variant	TP53	known	W53*	159G>A
1.6E+09	short-variant	FGF6	known	V127M	379G>A
1.6E+09	short-variant	GNAS	known	R201C	601C>T

1.6E+09	short-variant	CDKN2A	likely	V96fs*21	287_294delTGCTG(
1.6E+09	short-variant	KRAS	known	G12D	35G>A
1.6E+09	short-variant	PIK3CA	known	N345K	1035T>A
1.6E+09	short-variant	TP53	known	R175H	524G>A
1.6E+09	short-variant	TIPARP	known	R133Q	398G>A
1.6E+09	copy-number	ZNF217	known	-	-
1.6E+09	copy-number	GNAS	known	-	-
1.6E+09	copy-number	NKX2-1	known	-	-
1.6E+09	copy-number	ARFRP1	known	-	-
1.6E+09	rearrangeme	ALK	known	-	-
1.6E+09	short-variant	ERBB2	known	V659E	1976_1977TT>AG
1.7E+09	short-variant	TP53	likely	S303fs*49	907_908insCCACGA
1.7E+09	short-variant	EGFR	known	L858R	2573T>G
1.7E+09	short-variant	KRAS	known	G12V	35G>T
1.7E+09	short-variant	TP53	known	A69fs*54	205delG
1.7E+09	short-variant	EPHA3	known	V983M	2947G>A
1.7E+09	short-variant	EGFR	known	L747_P753>S	2240_2257delTAAG
1.7E+09	short-variant	TP53	likely	splice site 376-5_3	376-5_382delTACA(
1.7E+09	copy-number	CDKN2A	known	-	-
1.7E+09	copy-number	SOX2	known	-	-
1.7E+09	copy-number	CDKN2B	known	-	-
1.7E+09	copy-number	NKX2-1	known	-	-
1.7E+09	rearrangeme	BRIP1	likely	-	-
1.7E+09	copy-number	CDK4	known	-	-
1.7E+09	copy-number	MDM2	known	-	-
1.7E+09	copy-number	ERBB3	known	-	-
1.7E+09	rearrangeme	ALK	known	-	-
1.7E+09	short-variant	TP53	known	C176F	527G>T
1.7E+09	short-variant	FLT1	known	R593W	1777C>T
1.7E+09	short-variant	EGFR	known	E746_A750del	2235_2249delGGA/
1.7E+09	copy-number	EGFR	known	-	-
1.7E+09	copy-number	CDK6	known	-	-
1.7E+09	short-variant	TP53	known	V172F	514G>T
1.7E+09	short-variant	EGFR	known	E746_A750del	2235_2249delGGA/
1.7E+09	short-variant	KRAS	known	G12V	35G>T
1.7E+09	short-variant	TP53	known	C176G	526T>G
1.7E+09	copy-number	NKX2-1	known	-	-
1.7E+09	short-variant	TP53	known	R248W	742C>T
1.7E+09	short-variant	NF1	likely	K969fs*3	2905_2912delAAG/
1.7E+09	short-variant	EGFR	known	L747_P753>S	2240_2257delTAAG
1.7E+09	copy-number	MYC	known	-	-
1.7E+09	copy-number	CDKN2A	known	-	-
1.7E+09	copy-number	CDKN2B	known	-	-
1.7E+09	copy-number	EPHA3	known	-	-
1.7E+09	copy-number	EGFR	known	-	-
1.7E+09	copy-number	MTAP	known	-	-
1.8E+09	short-variant	BCOR	likely	K175fs*40	524_527delAACA
1.8E+09	short-variant	EGFR	known	E746_A750del	2235_2249delGGA/

1.8E+09	short-variant	TP53	known	R213Q	638G>A
1.8E+09	copy-number	EGFR	known	-	-
1.8E+09	copy-number	KEL	known	-	-
1.8E+09	copy-number	KDR	known	-	-
1.8E+09	copy-number	BRAF	known	-	-
1.8E+09	copy-number	EPHB4	known	-	-
1.8E+09	copy-number	ERBB2	known	-	-
1.8E+09	copy-number	CUL4A	known	-	-
1.8E+09	copy-number	RPTOR	known	-	-
1.8E+09	copy-number	CDK6	known	-	-
1.8E+09	copy-number	KIT	known	-	-
1.8E+09	short-variant	TP53	known	G244V	731G>T
1.8E+09	copy-number	CDK6	known	-	-
1.8E+09	short-variant	RNF43	likely	R343fs*100	1026_1027insT
1.8E+09	short-variant	TP53	known	Y220C	659A>G
1.8E+09	short-variant	CSF1R	likely	Q764*	2290C>T
1.8E+09	short-variant	EGFR	known	E746_A750del	2235_2249delGGA
1.8E+09	copy-number	CDKN2B	known	-	-
1.8E+09	copy-number	NKX2-1	known	-	-
1.8E+09	copy-number	CDKN2A	known	-	-
1.8E+09	copy-number	MTAP	known	-	-
1.8E+09	copy-number	NFKBIA	known	-	-
1.9E+09	short-variant	KIT	known	D816Y	2446G>T
1.9E+09	short-variant	ASXL1	known	E635fs*15	1900_1922del23
1.9E+09	short-variant	ERBB2	known	V777L	2329G>T
1.9E+09	short-variant	DNMT3A	likely	R598*	1792C>T
1.9E+09	short-variant	BCOR	likely	P405fs*35	1211_1212insA
1.9E+09	short-variant	CIC	likely	splice site 2887-40	2887-40_2970del1
1.9E+09	short-variant	KRAS	known	G12C	34G>T
1.9E+09	copy-number	KRAS	known	-	-
1.9E+09	copy-number	NKX2-1	known	-	-
1.9E+09	copy-number	BCL2L2	known	-	-
1.9E+09	copy-number	CDK4	known	-	-
1.9E+09	short-variant	TP53	known	R342*	1024C>T
1.9E+09	short-variant	EGFR	known	E746_A750del	2236_2250delGAA
1.9E+09	copy-number	EGFR	known	-	-
1.9E+09	short-variant	TP53	known	H179R	536A>G
1.9E+09	short-variant	MED12	known	D23Y	67G>T
1.9E+09	short-variant	EGFR	likely	L747_T751>Q	2239_2252TTAAGA
1.9E+09	copy-number	RB1	known	-	-
1.9E+09	copy-number	STK11	known	-	-
1.9E+09	copy-number	EGFR	known	-	-
1.6E+09	short-variant	EGFR	known	N771>GY	2311A>GGTT
1.6E+09	short-variant	TP53	known	H193L	578A>T
1.6E+09	copy-number	RB1	known	-	-
1.6E+09	short-variant	ATM	likely	N1094fs*14	3279_3282delCAAT
1.6E+09	short-variant	ERBB2	known	A775_G776insYVM	2324_2325insATAC
309634	short-variant	CHEK2	likely	splice site 444+1G	444+1G>A

309634	short-variant	TP53	known	R248Q	743G>A
309634	short-variant	U2AF1	known	S34F	101C>T

SV-COVERAGE	SV-PERCENT-READS
299	17.73
736	62.77
-	-
-	-
-	-
-	-
-	-
-	-
-	-
-	-
1847	80.45
516	53.68
-	-
-	-
-	-
497	23.94
412	24.76
-	-
-	-
518	63.9
778	32.78
735	16.73
437	17.39
-	-
-	-
-	-
-	-
1068	9.46
706	41.64
1549	34.93
-	-
607	45.8
659	37.18
556	48.56
-	-
1165	34.85
859	19.44
1306	37.29
629	23.37
769	35.24
-	-
729	27.02
705	15.18
567	6
727	37.83
734	32.97
743	1.21

	754	38.06
	713	37.59
	686	22.74
	774	13.82
	439	30.52
-	-	
	276	74.28
	582	74.05
-	-	
	543	46.78
	311	43.09
-	-	
-	-	
-	-	
	294	13.61
	201	13.93
-	-	
	673	21.4
	620	22.9
	280	8.93
-	-	
	361	44.32
	563	66.79
-	-	
	206	29.13
	627	16.27
	730	7.81
	1193	7.46
	1106	7.32
	915	1.97
-	-	
	953	28.12
	958	23.8
	1027	44.79
	545	7.71
	644	47.36
-	-	
-	-	
	1273	35.66
	440	15.45
	1165	41.55
-	-	
-	-	
-	-	
	432	10.88
	706	11.61
	977	11.57
	4838	81.5

982	37.37
920	21.2
-	-
-	-
801	19.1
779	3.34
998	14.43
-	-
-	-
-	-
-	-
677	33.08
539	50.09
540	7.41
-	-
-	-
-	-
-	-
-	-
618	27.02
586	31.23
-	-
-	-
-	-
-	-
-	-
363	59.78
365	56.99
770	46.23
4298	82.67
-	-
-	-
-	-
592	41.22
-	-
-	-
-	-
-	-
-	-
-	-
-	-
561	22.82
641	38.69
669	37.52
-	-
-	-
-	-
1197	12.2

903	15.5
694	39.48
764	47.12
628	15.76
1095	34.52
664	23.64
1188	49.75
-	-
-	-
-	-
961	27.68
965	26.42
936	15.28
897	24.53
875	24.69
108	12.04
487	51.75
734	16.62
296	32.09
-	-
1054	38.43
726	45.45
-	-
703	9.1
850	11.18
767	10.17
617	6.48
390	51.03
156	16.67
296	17.57
-	-
959	58.6
-	-
-	-
557	16.52
606	15.18
-	-
-	-
1149	23.24
951	36.38
-	-
844	30.81
655	49.01
1178	45.5
638	39.34
372	13.44
593	51.1
851	2

483	9.73
797	12.05
607	4.28
155	32.9
495	52.12
-	-
-	-
-	-
-	-
-	-
671	18.03
1615	50.22
1393	41.85
868	14.06
527	19.35
914	1.75
908	29.41
451	27.94
-	-
-	-
-	-
-	-
-	-
-	-
-	-
-	-
-	-
480	27.71
631	44.37
1799	75.43
-	-
-	-
290	12.41
547	16.82
826	22.52
668	34.88
-	-
356	72.19
380	71.32
5082	89.2
-	-
-	-
-	-
-	-
-	-
-	-
523	45.7
1490	48.19

	551	57.89
-	-	
-	-	
-	-	
-	-	
-	-	
-	-	
-	-	
-	-	
-	-	
-	-	
	999	13.71
-	-	
	895	25.92
	616	80.36
	641	56.63
	1639	68.4
-	-	
-	-	
-	-	
-	-	
-	-	
	1261	36.08
	1281	45.59
	852	35.68
	960	36.35
	790	20.13
	567	14.29
	2735	74.92
-	-	
-	-	
-	-	
-	-	
	684	28.65
	2995	75.29
-	-	
	465	38.92
	496	2.62
	2114	72.88
-	-	
-	-	
-	-	
	977	41.25
	1115	64.04
-	-	
	768	17.97
	852	18.78
	441	50.34

349	24.36
677	13.29
