# Supplementary figures and images for: Genomic landscape of lung cancer in the young
Source: Front Oncol. 2022 Sep 29;12:910117. doi: 10.3389/fonc.2022.910117 (PMC9575317; doi:10.3389/fonc.2022.910117)

## SUPPLEMENTARY FIGURES

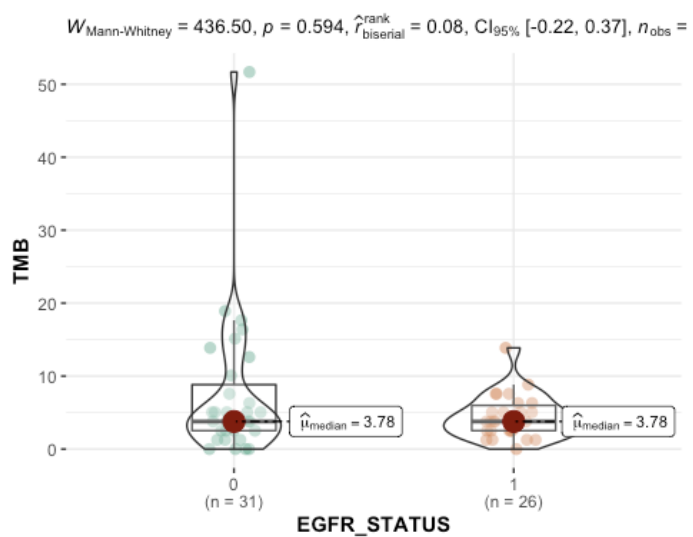

Figure S1,. TMB according the EGFR status regardless age group.

Supplement: Supplementary file 3 [file DataSheet_3.pdf]
